# Balancing risk: the influence of human disturbance and predators on roe deer activity patterns

**DOI:** 10.1093/cz/zoaf048

**Published:** 2025-08-05

**Authors:** Elisa Torretta, Giulia Ruffoni, Erika Bergantin, Eleonora Frigerio

**Affiliations:** Department of Earth and Environmental Sciences, University of Pavia, Via Ferrata 1, 27100 Pavia, Italy; Department of Veterinary Medicine, University of Sassari, Via Vienna 2, 07100 Sassari, Italy; Department of Earth and Environmental Sciences, University of Pavia, Via Ferrata 1, 27100 Pavia, Italy; Department of Earth and Environmental Sciences, University of Pavia, Via Ferrata 1, 27100 Pavia, Italy

**Keywords:** antipredator responses, behavioral plasticity, camera-trapping, *Capreolus capreolus*, northwestern Italy

## Abstract

Animal activity patterns are influenced by endogenous and exogenous factors, with human disturbance and predator presence playing a key role in fearful prey. This study explored diel activity patterns of the roe deer (*Capreolus capreolus*) across different study areas with varying levels of human disturbance and predator presence, specifically wolves (*Canis lupus*), located in northwestern Italy. We conducted seasonal camera-trapping sampling sessions for 2 years (2020–2022); we deployed 502 camera traps for a cumulative sampling period of 6,709.5 trapping days, and we collected 2,749 roe deer events. Roe deer exhibited generally bimodal diel activity patterns with peaks at dawn and dusk across all study areas, reflecting their crepuscular behavior. The consistency of the patterns can be attributed to biological constraints, such as mating and foraging needs, as observed during spring across all study areas, or to the absence of variations in perceived risks, as observed where human disturbance remains constant throughout the year. Interestingly, significant local and seasonal variations were also observed. Nocturnal activity varied seasonally and was influenced by landscape composition and predator presence, with lower nocturnal activity in modified landscapes during summer and autumn, likely due to favorable daytime conditions, and lower nocturnal activity in areas with stable wolf presence, particularly in winter, suggesting an antipredator response. Our results indicated that roe deer adapt their activity patterns to mitigate risks, demonstrating behavioral plasticity in response to the environment. This behavioral adaptability may be a key trait that facilitates the expansion of the species into highly anthropized landscapes, allowing roe deer to thrive in increasingly urbanized environments.

## Graphical Abstract

**Graphical Abstract:**
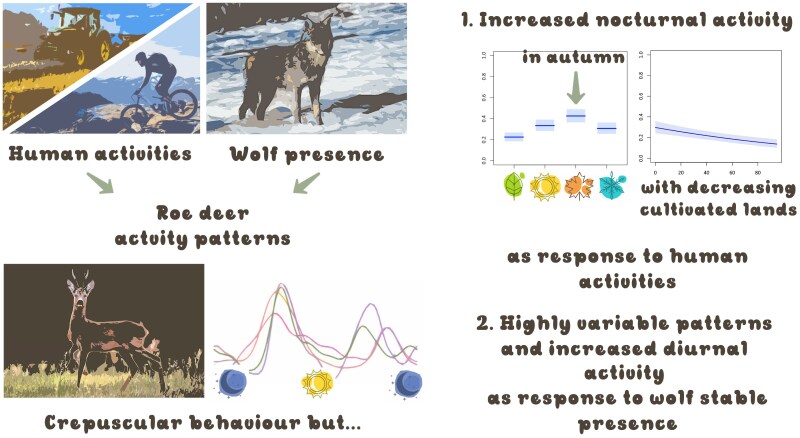

Many species cope with rapid environmental changes, such as those induced by human activity, through behavioral responses, with behavioral plasticity being a key trait that determines their success or failure in altered ecosystems (Sih et al. 2011). Prey species can perceive anthropogenic disturbances similarly to predation risks, prompting them to weigh trade-offs between avoiding perceived threats and engaging in fitness-enhancing activities, like foraging, parental care, or mating (Frid and Dill 2002; Gaynor et al. 2019). Fear, therefore, can significantly shape behavior (Brown et al. 1999; Laundré et al. 2010; Gaynor et al. 2019), triggering both reactive responses, such as flight (Stankowich 2008), and proactive responses (Creel 2018). These are more likely to emerge when animals encounter spatially and temporally predictable risks (Creel 2018; Riotte-Lambert and Matthiopoulos 2020), such as human activities, which are typically restricted to specific areas (e.g., villages, roads, and trails) and daytime hours (Courbin et al. 2022). For instance, increased nocturnality is a well-documented response to human disturbance (Gaynor et al. 2018; Procko et al. 2023). More broadly, adjustments along the temporal niche have been reported as common proactive responses to the perceived risks (Gaynor et al. 2018; Shamoon et al. 2018; Vicedo et al. 2023).

The diel activity patterns of animals (i.e., the distribution of activity throughout the daily cycle; hereafter, activity patterns) are influenced by a multitude of factors. Endogenous factors encompass physiological states and inherited traits, whereas exogenous factors such as weather conditions, duration of daylight, and human and predator presence also play crucial roles in shaping animal activity (Reimoser 2012). For wild ungulates, primary drivers of activity patterns include seasonal variations in resource abundance, weather, photoperiod, reproduction, and the presence of predators and humans (Di Bitetti et al. 2008; Podgórski et al. 2013) as key environmental factors. Additionally, ungulate activity is influenced to some degree by population density (Ramirez et al. 2021).

The roe deer (*Capreolus capreolus*), owing to its selective feeding strategy and relatively short bouts of rumination, is known to be a highly active animal (Cederlund 1989). The patterns of species activity mainly reflect various energy intake requirements, thus showing significant variability across seasons and times of day. Furthermore, activity patterns vary based on factors such as sex and age, as well as climate and other environmental conditions (Danilkin and Hewison 1996).

Regarding activity patterns, the roe deer typically exhibits 2 peaks of maximum activity approximately at dusk and dawn, with variations throughout the year, thus adapting to the photoperiod (Danilkin and Hewison 1996; Pagon et al. 2013; Stache et al. 2013; Bonnot et al. 2020).

Despite being primarily considered a crepuscular species, the roe deer can exhibit activity throughout the entire 24 h period (Pagon et al. 2013; Stache et al. 2013; Bonnot et al. 2020), with variations in intensity depending on the season. For example, daytime activity tends to increase in winter, whereas nighttime activity becomes more pronounced in summer, likely as a thermoregulation strategy in response to temperature variations (Pagon et al. 2013).

Activity patterns in roe deer can be altered in response to the perceived risk by shifting activity to less risky periods of the day; such temporal antipredator responses are not consistent across all conditions (Cusack et al. 2020), as several factors may influence them. For instance, Bonnot et al. (2020) observed high plasticity in roe deer diel activity patterns, allowing them to adapt to changes in risk levels over time. Roe deer exhibit a strong tendency to shift their activity toward nighttime hours when faced with even minimal anthropogenic disturbance. This adjustment is driven by the concentration of human activities during the day, which is further intensified during hunting seasons when the perceived risk increases (Bonnot et al. 2020). However, if nocturnal predators, such as large carnivores, are present, this behavioral strategy could increase the risk of predation. Consequently, a shift toward reducing nighttime activity and increasing daytime activity is required to mitigate these contrasting risk factors (Bonnot et al. 2020; Rossa et al. 2021). Therefore, the behavioral responses triggered by anthropogenic disturbance and predation risk can be conflicting, requiring careful balancing. As a result, in areas with high levels of human disturbance and predation pressure, roe deer may concentrate as much of their activity as possible into the least risky periods of the day.

Although the influence of predation and human disturbance on roe deer activity patterns has been studied, research explicitly addressing their combined effects remains limited (Bonnot et al. 2020). This gap is particularly evident in highly urbanized landscapes across Europe (Jasińska et al. 2021; Ruco et al. 2024), where both factors may exert significant and contrasting pressures on roe deer behavior. Our study provided a unique opportunity to examine roe deer under varying circumstances across different areas, applying a consistent methodology to assess whether previously observed patterns in their activity could be reliably repeated and better understood in light of human and predator pressures.

Our aim was to explore the responses of the roe deer to different levels of human disturbance and predation risk by analyzing species activity patterns to assess potential temporal avoidance as an antipredator strategy. We predicted that 1) in areas where predators were absent but human activity was high, roe deer would exhibit increased nocturnal activity as a response to avoid periods of peak human activity, particularly during the hunting seasons when perceived risk is increased (Bonnot et al. 2020); 2) conversely, in areas where predators, namely wolves (*Canis lupus*), were present and human activity was low, the response would be primarily determined by predation risk, prompting roe deer to avoid crepuscular and nocturnal hours (Bonnot et al. 2020; Rossa et al. 2021), thus avoiding periods when wolves are mainly active (Torretta et al. 2017a; Sunde et al. 2024; De Feudis et al. 2025); 3) in areas where both wolves and humans contributed to delineating the landscape of fear, roe deer responses would be expected to achieve a balance, resulting in activity patterns that reflect an interplay between the 2 constraints. However, identifying clear behavioral responses may be more complex in such contexts, as the combined effects of predation risk and human disturbance can induce conflicting outcomes. Nonetheless, because human activity is generally more predictable than predator movements, roe deer are expected to show more consistent adjustments in response to the anthropogenic disturbance.

## Materials and methods

### Study areas

**Figure 1:**
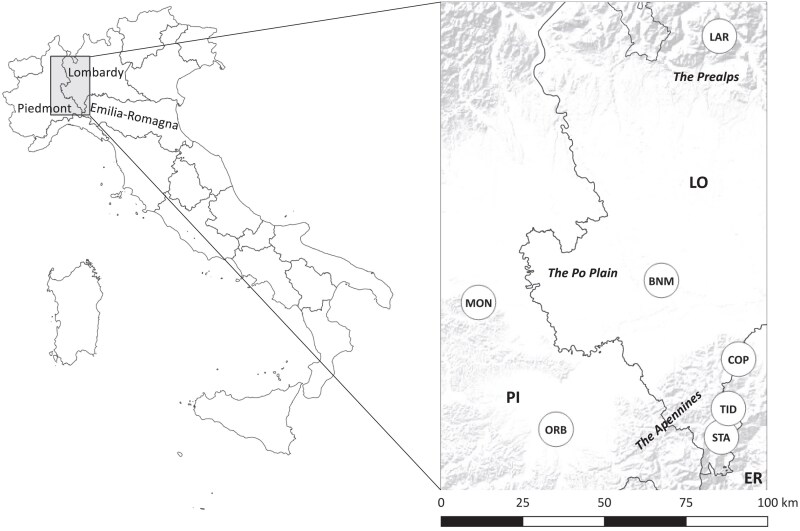
Location of the study areas in northwestern Italy (LO: Lombardy; PI: Piedmont; ER: Emilia-Romagna). Map created using QGIS version 3.3 (QGIS Development Team, 2025). Basemap: Esri Light Gray Canvas © Esri, HERE, Garmin, FAO, NOAA, USGS. Basemap accessed via XYZ Tiles using the following URL: https://server.arcgisonline.com/ArcGIS/rest/services/Canvas/World_Light_Gray_Base/MapServer/tile/{z}/{y}/{x}.

This research has been conducted in 7 study areas situated in northwestern Italy, spanning the regions of Lombardy, Piedmont, and Emilia-Romagna: Triangolo Lariano (LAR), Boschi Negri e Moriano (BNM), Basso Monferrato (MON), Torrente Orba (ORB), Colline Oltrepò Pavese (COP), Val Tidone (TID), and Valle Staffora (STA). The study areas exhibit notable diversity in landscape morphology and composition. In terms of topography, they are distributed from north to south, encompassing the Lombard Prealps (LAR), the Po Plain (BNM, MON, and ORB), and the Lombard Apennines (COP, TID, and STA). Considering landscape composition, 2 study areas (LAR and STA) are characterized by a predominant natural landscape with abundant woodlands. A mixed landscape with woodlands alternating with cultivated fields characterized the other 2 study areas (BNM and TID). Finally, the last 3 study areas (MON, ORB, and COP) are characterized by a predominantly agricultural, that is, modified, landscape with small and scattered patches of woodland (Figure 1; Supplementary Material S1: Supplementary Figure S1 and Table S1).

LAR is located in the Prealpine zone of the Lombardy region, an area characterized by the presence of many lakes. LAR has a predominant natural landscape with abundant woodlands (79.4% of the surface) mainly composed of mixed thermophilous species (e.g., *Ostrya carpinifolia*, *Acer* spp., *Fraxinus* spp., *Tilia* spp.), beech (*Fagus sylvatica*) and chestnut (*Castanea sativa*). Agricultural areas are mainly constituted of arable fodder crops and hay fields (9.5%) and are restricted to the valley bottoms. Main human activities are extensive farming, livestock (dairy production), and recreational activities, such as hunting and hiking.

BNM is located in the Po Plain along the Ticino River in the Lombardy region. It is characterized by a mixed landscape with an alternation of woodlands and cultivated lands: mesohygrophile and multilayered woodlands (*Quercus robur* and *Populus* spp.), which are the remnants of the native forest of the alluvial plain, and riparian woodlands (*Salix* spp. and *Alnus glutinosa*) represent a continuous canopy along the river, whereas arable lands (38.0%; mainly maize, other cereals, and paddies) are predominant among the agricultural patches. Main human activities are intensive farming (cereal production) and recreational activities, such as hiking and biking; the area is also frequented by dogs and their owners. BNM is largely included in the Natural Park “Valle del Ticino Lombardo Regional Park” (91.8% of the protected area). In this area, hunting is forbidden.

MON is a foothill area located along the Po River in the Piedmont region. MON lies in an intensive agricultural landscape, where crops, mainly maize (13.6%) and paddies (10.5%), are cultivated over large fields, and poplar plantations (15.2%) are widespread. Within this agricultural landscape, there is a floodplain natural area (462 ha), where riparian woodland is mainly composed of *Salix* spp., *Quercus* spp., and *Robinia pseudoacacia*. Main human activities are intensive farming (cereal production) and hunting. MON is partially included in the Natural Park “Po Piemontese Regional Park” (43.5% of protected area), where hunting is forbidden.

ORB is located in the Po Plain along the Orba Stream in the Piedmont region. ORB is characterized by an agricultural landscape made up of many small, diversified patches. Arable lands (65.0%) are allocated, for example, to maize and other cereals, alfalfa, vegetables, and sunflowers, and are alternated to hay fields (6.9%). Whereas woodlands, mainly composed of *Populus* spp. and *Salix* spp. or *R. pseudoacacia*, are restricted to the small almost continuous canopy along the Orba stream (12.9%). Main human activities are farming and hunting. A small portion of ORB is included in the Nature Reserve of the Orba Stream (14.2% of the protected area), where hunting is forbidden.

COP is a hilly area located in the Apennines between the Lombardy and Emilia-Romagna regions. It is characterized by vast vineyard cultivations (69.4%), whereas woodlands are represented by small isolated patches scattered among vineyards (6.5%). Main human activities are intensive farming (wine production) and hunting.

TID lies between the upper hills and the mountains of the Apennines between Lombardy and Emilia-Romagna regions. TID is characterized by a mixed landscape where woodlands are alternated with cultivated lands, mainly constituted of rotational crops (alfalfa and wheat or barley; 37.9%). Broad-leaved woodlands are predominant (37.9%) and composed of thermophilous species dominated by *Quercus pubescens* in association with *Quercus petraea, Quercus cerris*, *Fraxinus ornus, O. carpinifolia*, and *Acer campestre*. Shrublands are also widespread (13.0%). Main human activities are extensive farming, husbandry (mainly cattle), and, among recreational ones, hunting and motocross.

STA is a mountainous area located in the Apennines between Lombardy and Emilia-Romagna regions. Woodlands are predominant (70.1%): among broad-leaved species, beech (*F. sylvatica*) and chestnut (*C. sativa*) are widespread; among conifers, *Pinus nigra*, *Picea abies*, and *Larix decidua* are the main species. Alongside woodlands, well-structured shrublands are present (8.5%). Agricultural areas are mainly constituted of hay fields (17.9%). Main human activities are extensive husbandry (mainly cattle) and, among recreational ones, hunting and motocross.

In LAR, BNM, and MON, the wolf was absent or occasionally present during the study period, whereas in ORB, COP, TID, and STA, it was present with stable packs. In ORB, the pack was detected in 2020–2021 (Marucco et al. 2022); in COP, the pack was detected in 2015–2016, whereas in STA and TID, the presence of wolves was stable since the 1990s (Meriggi et al. 2020). In the 4 study areas, the roe deer represented the most consumed prey species by wolves (Marucco et al. 2022; Torretta et al. 2024).

Other wild ungulate species are the wild boar (*Sus scrofa*) present in every study area; the fallow deer (*Dama dama*) in BNM, MON, TID, and STA; the red deer (*Cervus elaphus*) in TID and STA; and the mouflon (*Ovis gmelini musimon*) in LAR. Considering other potential predators of roe deer, the red fox (*Vulpes vulpes*) is present in all study areas (Supplementary Material S1: Supplementary Table S1).

### Data collection

In each study area, seasonal camera-trapping sessions (winter: December to February, spring: March to May, summer: June to August, and autumn: September to November) were conducted over a 2-year sampling period from December 2020 to November 2022. We maintained camera traps in the field for a putative period of 120 days per seasonal session in each study area.

We subdivided each study area into sample squares using a 1.5 × 1.5 km grid (Supplementary Material S2: Supplementary Table S2.1). Within each sample square, 1 camera trap was randomly deployed during each sampling session. We adopted the sampling design named Tessellation Stratified Sampling, which ensures the homogeneous coverage of the area and, thus, allows a better distribution of random samples and increases their representativeness (Barabesi and Fattorini 2013). We selected the size of the sample squares based on our target species’ spatial ecology (i.e., home range and movements) and similarly to other studies (Rossa et al. 2021 [sample squares: 1 km × 1 km]; Donini et al. 2025 [sample squares: 1.5 km × 1.5 km]). Moreover, to reduce spatial correlation, we spaced the camera traps sufficiently apart to minimize the likelihood of capturing the same individual at multiple traps, following Marcon et al. (2019) who set the minimum distance between camera traps at 500 m (target species: roe deer; study area: Tuscany, Italy).

Camera traps were deployed and activated simultaneously during each seasonal session to ensure continuous monitoring 24 h a day throughout the sampling period.

We used 2 camera-trap models (Scout Guard SG520 and Apeman H55) with trigger speed ranging between 0.3 and 0.7 s. Camera traps were set to record 30-s videos with a minimum time delay between consecutive ones. They were mounted on trees and bushes mainly at a height of 50–100 cm (47.2% of deployed camera traps); alternative heights were chosen depending on vegetation and ground conditions (e.g., slope).

All recorded videos were carefully inspected to identify the captured species; consecutive videos regarding the same species were considered different events only if they were spaced at least 30 min apart to ensure capture independence (Kelly and Holub 2008; Monterroso et al. 2014; Torretta et al. 2016; Torretta et al. 2017b; Rossa et al. 2021). Whenever possible, recorded roe deer were classified by sex and age; we adopted a blind identification procedure and we discarded discordant records. Regarding age classification, 3 age classes were considered: juveniles (<1 year old), subadults (<2 years old), and adults (>2 years old). The date considered for the transition from 1 age class to the next has been identified as 1 June of each year. To classify the animals, body proportions, including overall size and shape, muscular development, relative head length, chest size, and neck thickness, were considered. Additionally, to estimate the age of females, the shape of the abdomen was observed (Mustoni et al. 2005; Varuzza 2019).

### Data analyses

The activity patterns were analyzed based on data obtained through camera trapping, specifically considering the dates and the times at which roe deer events were recorded. Activity patterns were estimated for each study area on a seasonal basis; data from the same season but different sampling sessions (e.g., spring 2021 and spring 2022) were combined. We considered a minimum of 25–30 detections as sufficient to provide representative estimates for the population (Lashley et al. 2018). To produce a model of circadian rhythms, we used the nonparametric method Kernel Density Estimation (Ridout and Linkie 2009), which considers camera-trapping events as representative samples of a continuous distribution over 24 h and employs a probability density function to produce a curve whose peaks are associated with a higher density of roe deer events, which can be interpreted as increased activity at the corresponding time of day. The Watson's test (*U*^2^) was used to evaluate the uniformity of obtained seasonal activity patterns (Lund et al. 2017).

We compared the different seasons within the same study areas to verify the consistency of species activity patterns on a local scale and among the different study areas within the same season. Specifically, a comparison of the activity patterns between areas differing in predator presence (stable vs. not stable) and landscape composition (natural vs. mixed vs. modified) was carried out. We tested the significance of observed differences by performing the Mardia–Watson–Wheeler test (*W*_g_) and post hoc Watson's 2-sample test (2-sample *U*^2^), applying the Bonferroni correction to *P*-values (Pewsey et al. 2013; Torretta et al. 2016); moreover, we quantified the similarities using the coefficient of overlap (Δ), which ranges from 0 (no overlap) to 1 (complete overlap; Ridout and Linkie 2009). Depending on the sample size (≤75 or > 75), we used either Δ_1_ or Δ_4_ (Meredith et al. 2024). Following Monterroso et al. (2014), we evaluated overlap values from pairwise comparisons categorizing overlap as low (≤50th percentile), moderate (51st to 75th percentile), or high (>75th percentile). Differences in the Δ values between seasons, predator pressures, and human disturbances were tested using nonparametric tests (i.e., Mann–Whitney test or Kruskal–Wallis test with Dunn test for pairwise comparisons).

Moreover, camera-trapping events were categorized into 4 periods of the diel cycle: day (from 1 h after sunrise to 1 h before sunset), night (from 1 h after sunset to 1 h before sunrise), dawn (1 h before to 1 h after sunrise), and dusk (1 h before to 1 h after sunset; Foster et al. 2013; Monterroso et al. 2014; Torretta et al. 2017b). The tendency toward diurnality or nocturnality of the roe deer was quantified using indices of diurnal and nocturnal activity (Clauss et al. 2021). These indices were calculated as proportions of the total events recorded during the day and the night compared with the overall events collected during camera-trapping sessions at each camera-trapping site; therefore, they can range between 0 and 1.

These 2 patterns (i.e., diurnality or nocturnality) can be considered complementary, as they reflect opposite but related adaptations in species activity patterns. Thus, we considered the nocturnal activity for subsequent analyses. A comparison at the seasonal level among the areas differing in predator presence (stable vs. not stable) and landscape composition (natural vs. mixed vs. modified) was carried out using nonparametric tests (i.e., Mann–Whitney test or Kruskal–Wallis test with Dunn test for pairwise comparisons).

We also performed generalized linear models (GLMs) where the index of nocturnal activity was modeled against the season (spring, summer, autumn, and winter), the landscape composition (natural, mixed, and modified), predator presence (stable and not stable), and the percentage cover of woodlands, natural open areas (i.e., natural grasslands, pastures, shrublands), cultivated lands, and protected surface (i.e., no hunting zones) within the sampling square of each camera-trapping site. Specifically, we applied beta regression, which assumes that all response values fall strictly between 0 and 1 (Ferrari and Cribari-Neto 2004). We generated a set of models using all possible combinations of uncorrelated variables, then we selected the best models based on AICc scores (models with ΔAICc ≤ 2; Burnham and Anderson 2002), ignoring redundant models (e.g., more complex versions of any simpler model).

We performed reported analyses using the “circular” (Lund et al. 2017), “overlap” (Meredith et al. 2024), “stats,” “MuMIn” (Bartoń 2023), and “betareg” (Cribari-Neto and Zeileis 2010) packages in R (R Core Team 2024).

## Results

### Sampling effort and collected data

In each study area, 8 seasonal camera-trapping sessions were carried out during the study period. Due to thefts and/or malfunctions of the camera traps, some camera-trapping sessions had durations different from the intended 120 days (Supplementary Material S2: Supplementary Table S2.2). The mean distance (±standard deviation) between camera traps during a sampling session was 1,098.2 ± 194.7 m (Supplementary Material S2: Supplementary Table S2.3). Thus, it is reasonable to assume that roe deer recorded at different camera trap sites corresponded to distinct individuals. Notably, roe deer exhibiting unique identifying features (e.g., males with a single antler or with abnormal antlers, females with antlers, and individuals with injuries or disabilities; *n* = 40) were consistently observed at singular camera trap sites, reinforcing this assumption.

Across all study areas and combining data from all 8 sampling seasons, a total of 2,749 roe deer events were recorded. The number of roe deer events recorded was sufficient to conduct the analyses (*n* ≥ 30) for each season in each study area (Table 1).

**Table 1:** Number of roe deer events recorded in the study areas located in northwestern Italy during the seasonal sampling sessions carried out from 2020 to 2022.

Study area	Sampling seasons								Total
	2020–2021				2021–2022				
	Winter	Spring	Summer	Autumn	Winter	Spring	Summer	Autumn	
LAR	71	33	46	18	39	57	46	59	369
BNM	81	87	152	43	109	90	93	78	734
MON	22	32	22	10	34	31	45	35	231
ORB	46	46	34	78	35	29	35	46	349
COP	24	41	48	51	26	59	48	16	313
TID	23	49	33	39	31	82	145	19	421
STA	11	47	32	25	27	45	106	40	327

### Activity patterns

The activity patterns of the roe deer showed a significant deviation from uniform distribution in almost all seasons in every study area, except for the winter season in LAR study area, the summer season in ORB study area, and the summer and autumn seasons in STA study area (Table 2).

**Table 2:** Results of Watson's test (*U*^2^) used to evaluate the uniformity of seasonal activity patterns.

Study area	Spring		Summer		Autumn		Winter	
	*U* ^2^	*P*	*U* ^2^	*P*	*U* ^2^	*P*	*U* ^2^	*P*
LAR	0.28	<0.010	0.22	<0.025	0.22	<0.050	0.09	>0.100
BNM	0.73	<0.010	0.45	<0.010	0.32	<0.010	0.36	<0.010
MON	0.42	<0.010	0.80	<0.010	0.26	<0.025	0.29	<0.010
ORB	0.68	<0.010	0.11	>0.100	0.58	<0.010	1.19	<0.010
COP	0.49	<0.010	0.41	<0.010	0.40	<0.010	0.78	<0.010
TID	0.54	<0.010	0.54	<0.010	0.27	<0.010	0.35	<0.010
STA	0.41	<0.010	0.12	>0.100	0.18	>0.050	0.51	<0.010

**Figure 2:**
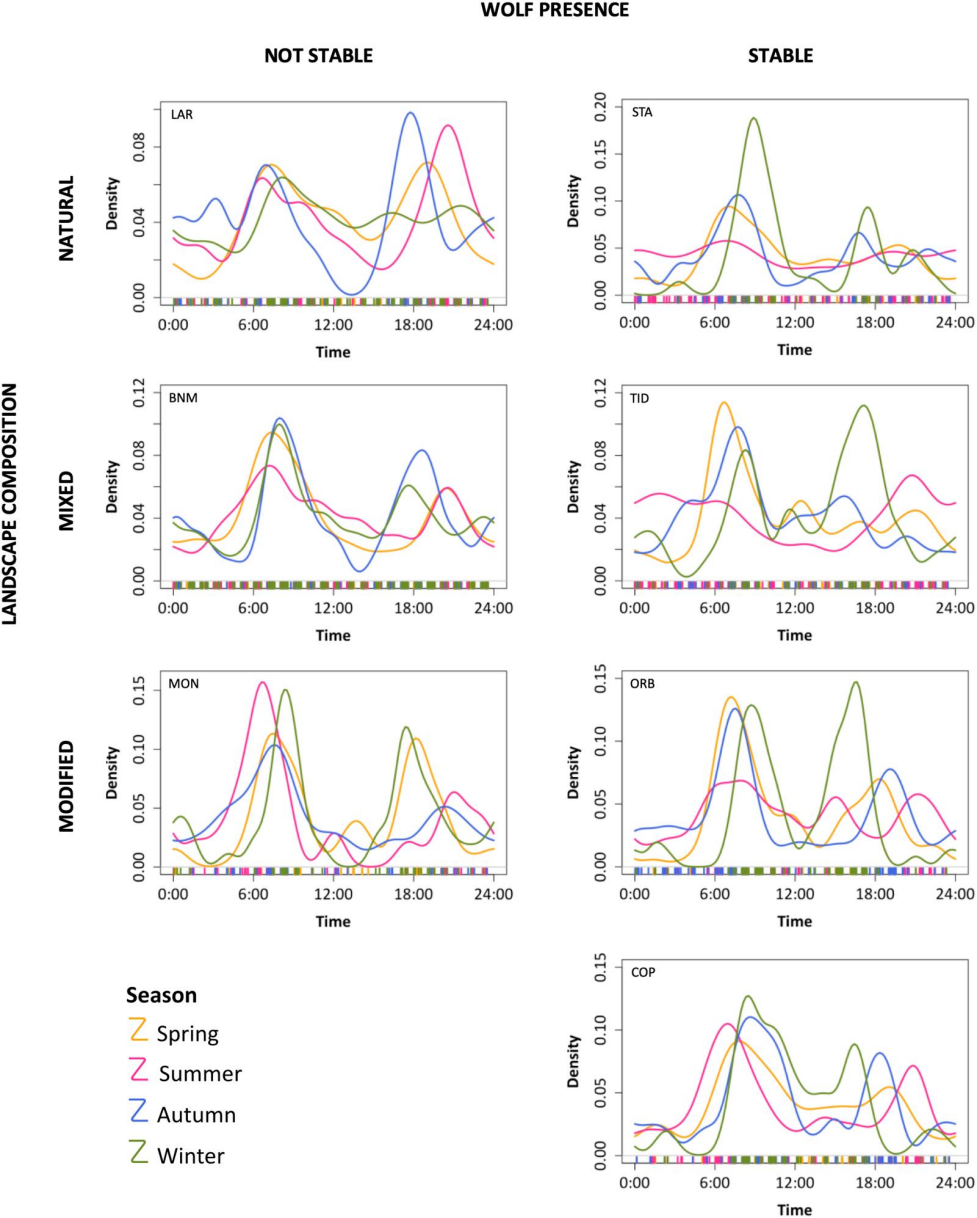
The activity patterns of the roe deer in northwestern Italy estimated for each season using the data collected from 2020 to 2022.

Generally, the roe deer showed nonuniform patterns with 2 main peaks of activity, at dawn and dusk hours, in every study area with seasonal differences (Figure 2).

**Figure 3:**
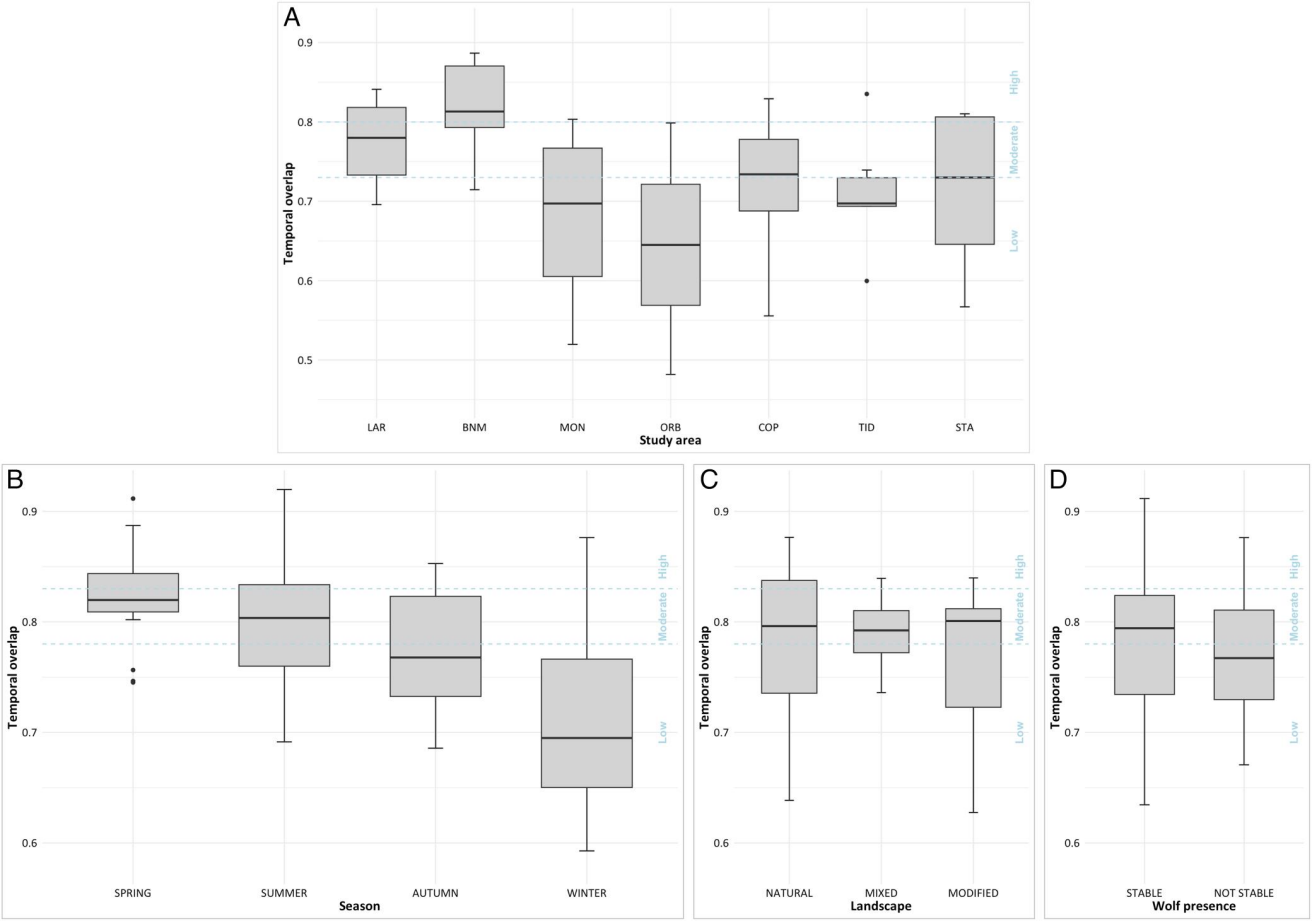
Temporal and spatial similarity of roe deer activity patterns in northwestern Italy quantified by the coefficient of overlap (Δ): (A) between seasons within each study area; (B) across study areas within the same season; (C) across areas differing in landscape composition; (D) across areas differing in predator presence.

Comparing activity patterns across different seasons revealed notable differences in most study areas, except for LAR and BNM, where the activity patterns remained consistent and uniform. The coefficient of overlap (Δ) showed moderate-to-high overlap between seasonal pairwise comparisons in these areas. In contrast, the remaining study areas exhibited high seasonal variability, as indicated by many significant pairwise comparisons and moderate-to-low seasonal overlap (Table 3 and Figure 3A).

**Table 3:** Results of Mardia–Watson–Wheeler test (*W*_g_) and post hoc Watson's 2-sample test (2-sample *U*^2^), with the Bonferroni correction to *P*-values, used to evaluate the uniformity of obtained activity patterns across seasons.

Study area	*W*_g_	*P*	Pairwise comparisons	*U* ^2^	*P*
LAR	10.74	0.097			
BNM	9.57	0.144			
MON	37.33	<0.001	SU vs. SP	0.71	<0.001
			SU vs. WI	0.70	<0.001
ORB	56.90	<0.001	WI vs. SP	0.63	<0.001
			WI vs. SU	0.45	<0.001
			WI vs. AU	1.60	<0.001
COP	32.98	<0.001	SU vs. SP	0.31	<0.01
			SU vs. AU	0.38	<0.01
			SU vs. WI	0.72	<0.001
TID	53.96	<0.001	SU vs. SP	0.74	<0.001
			SU vs. AU	0.51	<0.001
			SU vs. WI	0.77	<0.001
			SP vs. WI	0.32	<0.01
STA	22.65	<0.001	SU vs. SP	0.28	<0.01
			SU vs. WI	0.53	<0.001
			AU vs. WI	0.32	<0.01

In pairwise comparisons: SP = spring; SU = summer; AU = autumn; WI = winter.

The comparison of the activity patterns on a seasonal basis, that is, among different areas within the same season, revealed notable differences in all seasons except spring, where roe deer activity remained consistent and the Δ values showed moderate-to-high overlap. In contrast, summer, autumn, and especially winter showed high variability in activity patterns, with the Δ values indicating moderate overlap in summer and low overlap in autumn and winter (Table 4 and Figure 3B). The values of the coefficient of overlap were found to be different between seasons (*H* = 27.80, *df* = 3, *P* < 0.001); in particular, the temporal overlap was significantly higher and, therefore, the activity patterns were more uniform during spring and summer compared with winter (SP vs. WI: *P* < 0.001 and SU vs. WI: *P* = 0.001).

**Table 4:** Results of Mardia–Watson–Wheeler test (*W*_g_) and post hoc Watson's 2-sample test (2-sample *U*^2^), with the Bonferroni correction to *P*-values, used to evaluate the seasonal uniformity of obtained activity patterns.

Season	*W*_g_	*P*	Pairwise comparisons	*U* ^2^	*P*
Spring	20.07	0.066			
Summer	48.72	<0.001	LAR vs. MON	0.41	<0.001
			BMM vs. TID	0.65	<0.001
			BNM vs. MON	0.48	<0.001
			MON vs. TID	0.42	<0.001
			COP vs. TID	0.39	<0.001
Autumn	31.20	0.002	ORB vs. COP	0.51	<0.001
Winter	57.84	<0.001	LAR vs. ORB	0.54	<0.001
			LAR vs. COP	0.41	<0.001
			BNM vs. ORB	0.84	<0.001
			BNM vs. COP	0.53	<0.001
			MON vs. ORB	0.67	<0.001
			MON vs. COP	0.49	<0.001

Conversely, no significant differences emerged in the comparisons of the values of the coefficient of overlap between areas differing in predator presence (stable vs. not stable) and landscape composition (natural vs. mixed vs. modified; Figure 3C, D).

### Nocturnal activity

**Figure 4:**
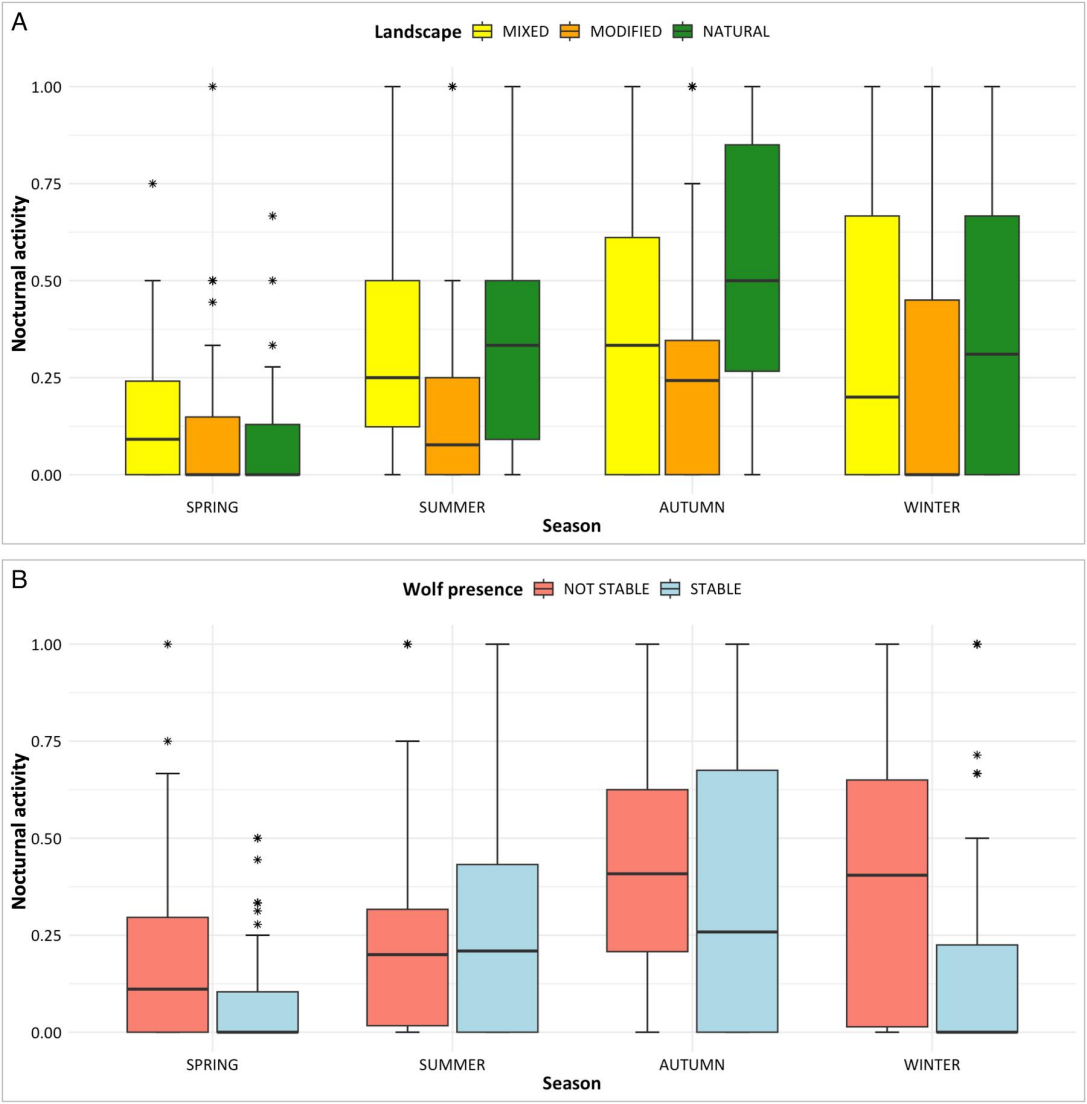
Seasonal variation in the nocturnal activity index of the roe deer across landscapes with different composition (A) and areas with different predator presence (B).

Considering the nocturnality index among the different landscapes within the same season, we observed significant seasonal variations during summer (*H* = 14.30; *P* = 0.001) and autumn (*H* = 8.49; *P* = 0.014). Specifically, during summer, lower nocturnality levels were observed in modified landscapes (MON, ORB, and COP) compared with natural (LAR and STA; *P* = 0.004) and mixed landscapes (BNM and TID; *P* = 0.005). Similarly, during autumn, lower nocturnality levels were observed in modified landscapes (MON, ORB, and COP) compared with natural ones (LAR and STA; *P* = 0.011; Figure 4A).

Moreover, significant variations also resulted when comparing the index across seasons within the same landscape. In natural landscapes (LAR and STA; *H* = 22.64; *P* < 0.001), lower nocturnality levels were observed during spring compared with summer (*P* = 0.008) and autumn (*P* < 0.001). Similarly, in mixed landscapes (BNM and TID; *H* = 12.56; *P* = 0.006), lower nocturnality levels were observed during spring compared with summer (*P* = 0.019) and autumn (*P* = 0.011). In modified landscapes (MON, ORB, and COP; *H* = 8.82; *P* = 0.032), lower nocturnality levels were observed during spring compared with autumn (*P* = 0.019; Figure 4A).

The nocturnal activity index was higher in areas with either an absence or occasional presence of wolves (LAR, BNM, and MON) during spring (*H* = 10.89; *P* = 0.001) and winter (*H* = 11.02; *P* = 0.001) compared with areas with a stable presence of wolves (ORB, COP, TID, and STA; Figure 4B).

We observed significant differences in areas where wolves are absent or occasionally present (LAR, BNM, and MON; *H* = 18.04; *P* < 0.001), where nocturnal activity levels were lower during spring compared with autumn (*P* = 0.001) and winter (*P* = 0.015). We also observed significant differences in areas where wolves were stable (ORB, COP, TID, and STA; *H* = 30.43; *P* < 0.001), where nocturnal activity levels were lower during spring compared with summer (*P* = 0.001) and autumn (*P* < 0.001); moreover, nocturnal activity levels were lower during winter compared with summer (*P* = 0.039) and autumn (*P* = 0.011; Figure 4B).

**Figure 5:**
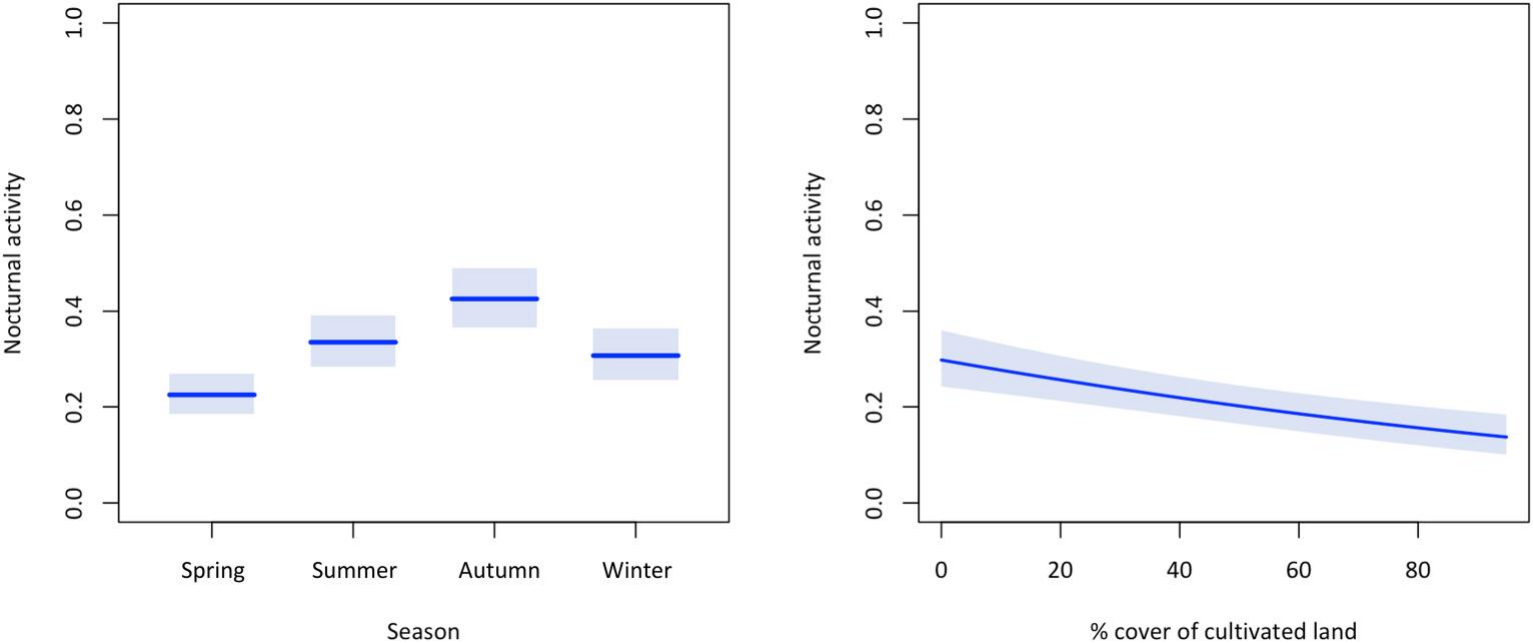
Factors influencing nocturnal activity levels of the roe deer in northwestern Italy. Response curves and 95% confidence intervals estimated through GLMs.

Considering top-ranked models describing nocturnal activity (*n* = 4), we considered only the first model, as the others were more complex versions of the best one (Supplementary Material S3: Supplementary Table S3). Nocturnal activity levels decreased with the increasing cover of cultivated lands and peaked during autumn (Table 5 and Figure 5).

**Table 5:** Factors influencing nocturnal activity levels of the roe deer in northwestern Italy. Estimates of model coefficients (*B*), standard errors (SE), 95% confidence intervals (95% CIs), and *P*-values are shown.

Variable	*B*	SE	95% CIs	*P*
(Intercept)	−0.859	0.143	−1.139, −0.578	<0.001
Season (summer)	0.549	0.167	0.221–0.876	0.001
Season (autumn)	0.938	0.175	0.596–1.280	<0.001
Season (winter)	0.421	0.171	0.086–0.756	0.014
% Cover of cultivated lands	−0.010	0.002	−0.014, −0.006	<0.001

Estimates of model coefficients (*B*), standard errors (SE), 95% confidence intervals (95% CIs), and *P*-values are shown.

## Discussion

The results obtained in this study underscored the general tendency toward bimodality of roe deer activity patterns, characterized by 2 peaks in activity around dusk and dawn periods. These findings confirmed the crepuscular behavior of the species (Danilkin and Hewison 1996; Pagon et al. 2013; Stache et al. 2013; Bonnot et al. 2020). Nevertheless, activity patterns exhibited notable variations at both local and seasonal scales.

Interestingly, in 2 study areas where wolves were absent or occasionally present (LAR and BNM), roe deer activity patterns exhibited uniformity, with no discernible seasonal differences detected. Assuming that variations in the circadian activity of roe deer reflect responses to changes in perceived risk levels, this condition of substantial uniformity throughout the year should therefore show a general lack of fluctuations in risk levels, which are mainly represented by human activities. In particular, in LAR study area, the landscape is uniformly occupied by woodlands, providing plentiful shelter sites, and the main sources of disturbance are outdoor activities (e.g., hiking and climbing) and hunting. Both activities have a strong, but opposite, seasonal variation. Outdoor activities take place mainly during the late spring and summer or winter seasons, whereas hunting is mainly done in autumn, but selective ungulate culling is also carried out during the other seasons with specific time frames allocated for different sexes and age classes. While acknowledging that the 2 forms of human activity have different impacts (indirect vs. direct disturbance), their common outcome is a near-continuous human presence across the landscape. Therefore, the activity may matter less than the consistent presence of humans (Grignolio et al. 2011), which can influence roe deer behavior year-round. In BNM study area, conversely, hunting is prohibited as it falls within a Protected Area. Therefore, human disturbance mainly stems from year-round recreational activities (e.g., hiking and biking) and agricultural fieldwork, which generally occurs within a few days of concentrated activity.

Among the other study areas, 4 of which have a stable wolf presence (ORB, COP, TID, and STA), there was greater variability in roe deer activity patterns during summer, autumn, and winter.

Summer is the season when anthropogenic disturbance from outdoor recreational activities, such as hiking and motocross, reaches its peak. These activities, especially off-road activities, can be a significant source of disturbance for wildlife because they can increase individual stress levels and disrupt normal behavior patterns and, thus, trigger antipredator responses. Interestingly, the results regarding summer activity patterns indicated a shift of the second peak of activity toward the nighttime hours, typically occurring between 9:00 PM and midnight, and an overall increase in nocturnal activity between the 2 peaks of higher activity, that is, at dusk and dawn. This trend toward a more pronounced nocturnal activity was also confirmed by the results of the nocturnal activity index, which, notably for mixed and natural landscapes, showed a significantly higher value. In fact, in some of the areas within these landscapes (e.g., TID and STA), the aforementioned activities are widely practized and not subject to any control or regulation. Pagon et al. (2013) attributed the pronounced nocturnal activity observed in summer to the need for thermoregulation. Although thermoregulation is a highly plausible explanation, if it were the primary factor determining roe deer activity patterns, we would have expected to observe more pronounced differences between the plain study areas, where summer temperatures are warmer, and the mountainous ones.

Concerning roe deer activity patterns during autumn, a noteworthy observation is the heightened nocturnal activity detected in LAR, ORB, TID, and STA study areas. These are all characterized by the beginning of the hunting season occurring in the same period. Indeed, driven hunting with hounds, which is the most impactful hunting activity, has been shown to modify the behavior of deer species in several ways, including the increase in nocturnal activity (Kilgo et al. 1998; Grignolio et al. 2011). Bonnot et al. (2020), in particular, observed that nocturnal activity can be adaptative and reflects roe deer response to the perceived risk of predation, even if is associated with human disturbance (especially hunting).

Even during winter significant variability in activity patterns was observed. In some study areas, for example, ORB and TID, the second peak of activity, occurring at dusk and encompassing the preceding hours leading up to it, was notably pronounced. Human presence during winter is generally minimal, both in terms of recreational activities and agricultural practices. However, besides human disturbance, climate and weather conditions also play a role in influencing roe deer activity patterns during the adverse season (Danilkin and Hewison 1996; Pagon et al. 2013). Decreased nighttime activity during winter has previously been observed and was believed to be associated with environmental factors (Pagon et al. 2013). In colder months, roe deer may increase daytime activity to search for food when temperatures are slightly warmer, although they still tend to exhibit crepuscular behavior.

Considering the study areas where these seasonal differences in activity patterns were most pronounced, it is notable that the 4 areas exhibiting the greatest variability (i.e., ORB, COP, TID, and STA) are those where the presence of wolves is stable. Human activities create a well-defined landscape of fear, as they are easily predictable for animals (e.g., agricultural disturbance is linked to cultivated fields and, thus, is spatially predictable), prompting proactive responses from prey, whereas the landscape of fear due to large carnivores may be more difficult for prey to anticipate (Bonnot et al. 2020). Interestingly, Crawford et al. (2021) observed increased heterogeneity in white-tailed deer (*Odocoileus virginianus*) behavior as a response to predator presence. Therefore, the antipredator responses to predation risk enacted by deer may be more temporally variable, particularly in landscapes where both human and carnivore pressures interact. Indeed, deer may experience significantly higher stress levels where human disturbances, exemplified by hunting harvests, roads, and built-up areas, are predominant (Zbyryt et al. 2018).

Interestingly, during spring the activity patterns of the roe deer remained consistent and uniform across the study areas. During this season, the patterns clearly exhibited the 2 typical peaks of activity, at dawn and dusk, along with moderate daytime activity and reduced nighttime activity. This pattern, observed in all areas, is linked to the biological and ecological characteristics of the species and is likely minimally influenced by landscape structure, human activities associated with it and other recreational activities, as well as wolf predation activity. Spring provides abundant easily digestible vegetation, which is essential for roe deer to regain body condition after the winter months. As rumination takes up less time than grazing, they spend a significant amount of time foraging for fresh grasses, herbs, and other plant matter (Danilkin and Hewison 1996). Moreover, during spring, roe deer engage in various activities that serve as a preparation for the summer rut. Bucks, in particular, focus on establishing and reinforcing their territories by marking them, increasing vocalizations, and engaging in low-level conflicts with rivals. These behaviors contribute to the conditions for the more intense competition of the rut, which takes place in summer and determines access to females for mating (Danilkin and Hewison 1996). Therefore, regarding the spring season, it seems that roe deer activity patterns are more regulated by endogenous factors rather than exogenous ones.

In general, the nocturnal activity of roe deer appears to be closely linked to seasonal variations, as previously discussed, as well as landscape composition and predator presence.

The lower nocturnality levels observed in modified landscapes, compared with natural and mixed landscapes, particularly during summer and autumn, suggest that human-altered landscapes may offer seasonal conditions that allow for increased daytime activity. Interestingly, a significant relationship between nocturnal activity levels and the abundance of cultivated lands has been detected. Cultivated areas not only offer plentiful and concentrated food resources but also provide suitable spaces conducive to daytime activities, potentially reducing predation risk for roe deer. Indeed, these spaces may offer both visibility, freedom of movement, and adequate cover, allowing roe deer to efficiently scan their surroundings for potential threats or predators while engaging in daytime activities such as feeding, grooming, and social interactions. The observed pattern regarding the nocturnal activity levels aligns with findings from other studies. For instance, Bonnot et al. (2013) demonstrated that roe deer may access open areas during the daytime, both as a source of food and cover, particularly in summer when crops are abundant in the fields and can also provide hiding cover.

Regarding predator presence, the higher nocturnal activity levels observed in areas with either absence or occasional presence of wolves, particularly during spring and winter, suggest that roe deer likely adjust their activity as an antipredator strategy. Although the shift toward diurnal activity in spring is likely driven by biological factors, and in winter, it may be influenced by lower temperatures (see above), adopting a diurnal activity pattern could also represent a response to predation risk. Deer demonstrate a remarkable ability to fine-tune their antipredator behavior based on the presence or absence of predatory threats on the landscape, especially during nonbreeding periods when even low levels of disturbance can trigger behavioral adjustments (Wiskirchen et al. 2022). Diurnality in the presence of natural predators is a well-known antipredator strategy in roe deer (Bonnot et al. 2020), allowing for the exploitation of the reduced threat from crepuscular and nocturnal predators while maximizing the ability to detect and respond to danger.

In summary, this study explored the behavioral adaptability of the roe deer in a human-dominated environment where its main predator, that is, the wolf, is rapidly expanding. Our results confirmed the crepuscular behavior of roe deer, with distinct activity peaks around dawn and dusk. However, notable seasonal variations were observed, influenced by both endogenous (biological) and exogenous factors, including human-induced disturbance and predator presence. Overall, our predictions were largely supported: 1) while hunting and recreational activities increased nocturnal activity in some study areas, this effect was not universal. In certain cases, roe deer maintained stable activity patterns despite human presence, suggesting that additional factors such as habitat composition and availability of shelter may play a role in shaping their responses. In particular, human-modified landscapes appeared to facilitate greater daytime activity, potentially reducing the need for nocturnal adjustments; 2) roe deer generally avoided nocturnal hours in response to wolf presence, supporting the hypothesis that predation risk influences their activity patterns. However, this response varied across seasons and landscapes, likely due to interactions between predation risk and other ecological factors. Notably, increased nocturnality was observed in summer and autumn, likely as an adaptive response to both human disturbances and predator avoidance, whereas in winter, diurnal activity increased, possibly due to colder temperatures and reduced human presence; 3) in areas where both wolves and human disturbances were present, roe deer exhibited complex behavioral responses, balancing the predictability of human-related threats with the more variable nature of predation risk. These responses were not only spatially dependent (based on landscape structure) but also temporally dynamic, shifting across seasons in response to fluctuating risk levels (Supplementary Material S4: Supplementary Table S4).

## Supplementary Material

zoaf048_Supplementary_Data
