# Supplementary material for: Balancing risk: the influence of human disturbance and predators on roe deer activity patterns
Source: Curr Zool. 2025 Aug 5;72(3):341–53. doi: 10.1093/cz/zoaf048 (PMC13290407; doi:10.1093/cz/zoaf048)
Supplement: zoaf048_Supplementary_Data [file zoaf048_supplementary_data.docx]

**Balancing risk: The influence of human disturbance and predators on roe deer activity patterns**

Elisa Torretta, Giulia Ruffoni, Erika Bergantin, Eleonora Frigerio

Corresponding author:

Dr Elisa Torretta

torretta.elisa@gmail.com

https://orcid.org/0000-0002-4773-6977

**Supplementary materials**

1. **Study areas**

Figure 1 - Land cover types (derived from Corine Land Cover 2018) characterizing the portion of north-western Italy encompassing the study areas

Table 1 - Main characteristics of the study areas

|  | **LAR** | **BNM** | **MON** | **ORB** | **COP** | | **TID** | | **STA** |
| --- | --- | --- | --- | --- | --- | --- | --- | --- | --- |
|  | **Triangolo Lariano** | **Boschi Negri e Moriano** | **Basso Monferrato** | **Torrente Orba** | **Colline**  **Oltrepò Pavese** | | **Val Tidone** | | **Alta Valle Staffora** |
| **Region** | LO | LO | PI | PI | LO - ER | | LO - ER | | LO - ER |
|  | LO: Lombardy; PI: Piedmont; ER: Emila-Romagna | | | | | | | | |
| **Elevation range (m a.s.l.)** | 200-1100 | < 100 | 100-350 | 110-145 | 130-410 | | 385-860 | | 550-1500 |
| **Range of annual temperature (°C)** | 2-19 | 1-23 | 2-23 | 3-23 | 2-23 | | 1-21 | | 1-19 |
| **Range of annual rainfall (mm)** | 51-141 | 41-105 | 41-94 | 32-125 | 37-105 | | 52-139 | | 64-188 |
| **Kind of landscape** | Natural | Mixed | Modified | Modified | Modified | | Mixed | | Natural |
| **Land cover classes:** |  |  |  |  |  | |  | |  |
| Urban areas | 8.3% | 3.3% | 7.1% | 5.3% | 7.4% | | 4.6% | | 2.2% |
| Cultivated lands: |  |  |  |  |  | |  | |  |
| arable lands | - | 19.3% | 13.6% | 65.0% | 10.7% | | 30.0% | | 11.6% |
| paddies | - | 18.7% | 10.5% | - | - | | - | | - |
| permanent crops | 0.1% | 0.4% | 2.7% | - | 69.6% | | 1.5% | | 0.5% |
| Woodlands: |  |  |  |  |  | |  | |  |
| broad-leaved woodlands | 64.4% | 26.6% | 26.6% | 12.9% | 6.5% | | 37.9% | | 53.4% |
| coniferous woodlands | 0.7% | - | - | - | - | | 2.0% | | 12.6% |
| mixed woodlands | 14.3% | - | - | - | - | | 1.4% | | 4.1% |
| Natural grasslands and pastures | 9.3% | 1.3% | 7.0% | 7.8% | 2.0% | | 7.9% | | 6.3% |
| Shrublands | 1.4% | 3.8% | 4.9% | 2.6% | 3.1% | | 13.0% | | 8.5% |
| Poplar plantation and reforestations | - | 16.6% | 15.3% | 0.9% | 0.7% | | 0.2% | | 0.4% |
| Areas without vegetation cover | 0.0% | 3.4% | 0.5% | 1.7% | - | | 1.0% | | 0.4% |
| Water bodies | 1.1% | 6.8% | 11.9% | 3.9% | - | | 0.4% | | - |
| **Wild ungulates community composition** | Cc,  Om, Ss | Cc,  Dd, Ss | Cc,  Dd, Ss | Cc,  Ss | Cc,  Ss | | Cc,  Dd, Ce, Ss | | Cc,  Dd, Ce, Ss |
|  | Cc: roe deer; Dd: fallow deer; Ce: red deer; Om: Mouflon; Ss: Wild boar | | | | | | | | |
| **Potential predators** | Vv | Vv | Vv | Vv; Cl | Vv; Cl | | Vv; Cl | | Vv; Cl |
|  | Vv: red fox; Cl: wolf | | | | | | | | |
| **Human density (inhab. km^-2^)** | 229.8 | 145.7 | 39.4 | 57.2 | | 91.0 | | 25.9 | 12.5 |
| **Hunting activity (Y/N)** | Yes | No | Yes (outside PA) | Yes (outside PA) | | Yes | | Yes | Yes |
| **% Protected Area (PA)** | - | 91.8 | 43.5 | 14.2 | | - | | - | - |

1. **Sampling effort**

Table 2.1 - Details on the sampling grid used for roe deer camera trapping in north-western Italy from 2020 to 2022

| **Study area** | **N° of sample squares** | **Total area (km^2^)** |
| --- | --- | --- |
| LAR | 9 | 20.25 |
| BNM | 12 | 27.00 |
| MON | 9 | 20.25 |
| ORB | 8 | 18.00 |
| COP | 8 | 18.00 |
| TID | 9 | 20.25 |
| STA | 8 | 18.00 |

Table 2.2 - The sampling effort of camera trapping expressed as mean (± SD) duration (on the first line) and total trapping days (on the second line) during the seasonal sessions in north-western Italy from 2020 to 2022

| **Study area** |  | **Sampling seasons** | | | | | | | | |
| --- | --- | --- | --- | --- | --- | --- | --- | --- | --- | --- |
|  |  | **2020-2021** | | | |  | **2021-2022** | | | |
|  |  | **Winter** | **Spring** | **Summer** | **Autumn** |  | **Winter** | **Spring** | **Summer** | **Autumn** |
| LAR |  | 16.4 ± 1.6  114.5 | 11.8 ± 6.1  107 | 14.8 ± 0.4  133 | 11.3 ± 2.8  113 |  | 19.6 ± 0.4  176 | 11.8 ± 4.6  94 | 15.0 ± 0.0  105 | 17.4 ± 0.5  157 |
| BNM |  | 13.0 ± 0.0  156 | 8.1 ± 2.3  97 | 14.0 ± 0.0  140 | 11.6 ± 1.7  105 |  | 16.3 ± 1.1  147 | 11.7 ± 3.9  105 | 13.9 ± 0.2  126 | 16.0 ± 0.0  144 |
| MON |  | 18.4 ± 3.3  129 | 13.2 ± 4.3  132 | 13.0 ± 0.0  117 | 12.9 ± 2.5  116 |  | 14.1 ± 0.3  127 | 9.4 ± 5.5  76 | 15.1 ± 1.7  136 | 21.0 ± 0.0  189 |
| ORB |  | 16.0 ± 5.8  128 | 9.2 ± 4.3  74 | 14.1 ± 3.8  113 | 13.9 ± 2.5  111 |  | 12.3 ± 3.0  99 | 12.5 ± 4.3  100 | 10.5 ± 4.4  84 | 16.0 ± 0.0  128 |
| COP |  | 14.3 ± 1.4  114 | 11.8 ± 4.2  95 | 12.6 ± 3.7  113 | 15.3 ± 3.8  138 |  | 8.3 ± 5.6  66 | 15.2 ± 1.9  107 | 15.0 ± 0.0  120 | 15.0 ± 0.0  120 |
| TID |  | 8.8 ± 3.7  79 | 13.0 ± 0.2  130 | 14.9 ± 0.2  135 | 14.8 ± 0.2  134 |  | 13.6 ± 4.1  122 | 13.3 ± 1.6  120 | 16.0 ± 0.0  144 | 15.9 ± 0.2  144 |
| STA |  | 13.4 ± 2.1  107 | 15.3 ± 0.3  122 | 9.9 ± 5.4  79 | 19.1 ± 0.3  153 |  | 13.4 ± 1.7  107 | 16.0 ± 7.7  128 | 15.3 ± 1.0  122 | 17.4 ± 0.7  139 |

Table 2.3 - Mean distance (± SD) expressed in meters between camera-trap sites during each seasonal session carried out in north-western Italy from 2020 to 2022

| **Study area** |  | **Sampling seasons** | | | | | | | | |
| --- | --- | --- | --- | --- | --- | --- | --- | --- | --- | --- |
|  |  | **2020-2021** | | | |  | **2021-2022** | | | |
|  |  | **Winter** | **Spring** | **Summer** | **Autumn** |  | **Winter** | **Spring** | **Summer** | **Autumn** |
| LAR |  | 1099.0 ± 438.9 | 760.5 ± 503.3 | 1009.6 ± 221.0 | 684.9 ± 433.0 |  | 1164.0 ± 417.5 | 1390.9 ± 233.9 | 1244.8 ± 276.7 | 969.2 ± 435.3 |
| BNM |  | 962.5 ± 244.9 | 1085.5 ± 286.9 | 1122.5 ± 226.2 | 867.0 ± 245.3 |  | 1138.4 ± 274.3 | 1419.0 ± 366.0 | 811.5 ± 267.0 | 817.1 ± 263.9 |
| MON |  | 1468.9 ± 446.4 | 1184.9 ± 331.7 | 936.2 ± 347.3 | 1379.8 ± 584.4 |  | 1103.1 ± 531.2 | 1122.9 ± 191.6 | 1025.3 ± 430.8 | 973.7 ± 600.7 |
| ORB |  | 882.8 ± 426.3 | 1295.6 ± 317.6 | 1189.1 ± 457.5 | 1030.4 ± 436.8 |  | 1370.0 ± 251.7 | 1351.9 ± 224.1 | 1160.7 ± 636.0 | 1123.0 ± 466.6 |
| COP |  | 719.1 ± 418.8 | 861.0 ± 337.9 | 1164.4 ± 315.1 | 922.5 ± 273.3 |  | 838.7 ± 358.6 | 1157.6 ± 62.7 | 1354.7 ± 432.7 | 918.5 ± 360.8 |
| TID |  | 1025.2 ± 172.1 | 1105.8 ± 254.8 | 1138.1 ± 385.6 | 1174.8 ± 420.8 |  | 906.5 ± 510.2 | 1083.3 ± 198.9 | 1244.9 ± 344.5 | 1214.9 ± 509.7 |
| STA |  | 1092.5 ± 750.8 | 961.8 ± 229.1 | 1568.8 ± 208.0 | 1205.2 ± 254.3 |  | 1008.3 ± 94.0 | 1247.5 ± 75.2 | 1140.5 ± 459.5 | 1299.1 ± 379.3 |

1. **Nocturnal activity**

**Model selection for factors influencing nocturnal activity levels of the roe deer in north-western Italy**

Table 3 - The top-ranked models are shown (Δ AICc ≤ 2); for each model, the variables, the number of parameters, AICc, ∆AICc and standardized weight are reported.

| **Model** | **Variables** | **K** | **logLik** | **AICc** | **Δ AICc** | **Weight** |
| --- | --- | --- | --- | --- | --- | --- |
| **Best** | **Season + % cover of cultivated lands** | **6** | **548.89** | **1085.57** | **0.00** | **0.40** |
| 2^nd^ | Season + % cover of cultivated lands + % cover of protected areas | 7 | 549.31 | 1084.34 | 1.23 | 0.22 |
| 3^rd^ | Wolf presence + Season + % cover of cultivated lands | 7 | 549.21 | 1084.15 | 1.42 | 0.20 |
| 4^th^ | Season + % cover of cultivated lands + % cover of natural open areas | 7 | 549.17 | 1084.06 | 1.51 | 0.19 |

1. **Summary of main results**

Table 4 - Roe deer activity patterns under different conditions

|  | **Condition** | **Roe deer activity patterns** | **Key findings** |
| --- | --- | --- | --- |
| **Wolf presence** | Stable | High variability (especially in summer, autumn, winter) and increased diurnal activity | Suggests anti-predator strategies and flexible temporal adjustments |
|  | Absent or occasional | Uniform activity patterns year-round and/or increased nocturnal activity | Reflects stable or predictable risk levels, mainly linked to diurnal human disturbance |
| **Landscape** | Modified landscapes | Increased diurnal activity, particularly during autumn, summer, and winter | During autumn and summer, cultivated lands offer food and cover, supporting safer daytime activity |
|  | Natural or mixed landscapes | Decreased diurnal activity | In structurally more homogeneous landscapes, behavioural responses tend to be driven more by other factors |
| **Season** | Spring | Consistent bimodal pattern (dawn and dusk peaks) | Driven by biological needs (e.g., foraging, reproduction) |
|  | Summer | Increased nocturnal activity | Response to heat stress and high diurnal human disturbance (e.g., recreational activities) |
|  | Autumn | Increased nocturnal activity | Linked to hunting pressure |
|  | Winter | Decreased nocturnal activity | Influenced by thermoregulation needs and reduced human pressure |
